# An Ounce Minnie Ball Imbedded in the Brain Fifteen Years Successfully Removed

**Published:** 1878-11-20

**Authors:** O. M. Doyle

**Affiliations:** Georgia


### AN OUNCE MINNIE BALL IMBEDED IN THE BRAIN FIFTEEN YEARS, SUCCESSFULLY REMOVED.

BY O. M. DOYLE, M. D. OF GA.

Mike Kemp, of Franklin county, Ga., a soldier in the Confederate army, received during the Tennessee campaign in 1863, a gunshot wound about the middle of the forehead, which his friends thought fatal as a portion of his brains popped out at the opening of the wound However, he rallied, came home, and after a time, attained to comparative good health, which continued up to last spring; at which time, spasms to which he had been subject occasionly, became more frequent and more severe.

On the first of July, of the present year, I was summoned to assist his regular physician, Dr. J. A. Johns, at which time the patient was found feeble and irritable, tending to fever and having from one to two severe convulsions each day. Under these circumstances, it was determined to operate at once.

We attempted to anæsthetize him, but after a thorough effort with both ether and chloroform failed to produce more than analgesia; and could not keep him that far under the influence all the time required for the operation.

After making a free crucial incision over the site of injury and dissecting back the integuments to the bone, the original opening made by the ball through the bone was found to be filled with very tough cartilagenous growth—being apparently an effort of nature to *supplementary* callous. The entire removal of this growth exposed the base of the ball, just past the inner plane of the bone through which it had passed—the whole of the ball being imbedded in the substance of the brain. The opening through the bone was now enlarged by carefully trimming with sharp instruments until it was thought to be sufficient for the exit of the ball, which was seized by passing a slender but strong forcep, such as are used in the extraction of the roots of teeth, and after securing the base of the ball sufficient pressure was used to force the points of the instrument into the lead, thus diminishing the diameter so as to favor the exit of the instrument and ball. The removal was accomplished without further trouble. There was but little hemorrhage from the inside of the cranium. The entire cavity in the brain where the ball had been for fifteen years, was visible, and pulsation of the brain was easily seen at a distance of ten or fifteen feet. He made a rapid recovery under ordinary treatment for such condition. Is now quite well.

NOTE.—Should have stated that the ball had a large spicula of bone imbeded in its apex with a sharp angle projecting.

